# Mechanism of coupling drug transport reactions located in two different membranes

**DOI:** 10.3389/fmicb.2015.00100

**Published:** 2015-02-24

**Authors:** Helen I. Zgurskaya, Jon W. Weeks, Abigail T. Ntreh, Logan M. Nickels, David Wolloscheck

**Affiliations:** ^1^Department of Chemistry and Biochemistry, University of OklahomaNorman, OK, USA

**Keywords:** Gram-negative bacteria, antibiotic resistance, drug efflux, periplasmic membrane fusion proteins

## Abstract

Gram- negative bacteria utilize a diverse array of multidrug transporters to pump toxic compounds out of the cell. Some transporters, together with periplasmic membrane fusion proteins (MFPs) and outer membrane channels, assemble trans-envelope complexes that expel multiple antibiotics across outer membranes of Gram-negative bacteria and into the external medium. Others further potentiate this efflux by pumping drugs across the inner membrane into the periplasm. Together these transporters create a powerful network of efflux that protects bacteria against a broad range of antimicrobial agents. This review is focused on the mechanism of coupling transport reactions located in two different membranes of Gram-negative bacteria. Using a combination of biochemical, genetic and biophysical approaches we have reconstructed the sequence of events leading to the assembly of trans-envelope drug efflux complexes and characterized the roles of periplasmic and outer membrane proteins in this process. Our recent data suggest a critical step in the activation of intermembrane efflux pumps, which is controlled by MFPs. We propose that the reaction cycles of transporters are tightly coupled to the assembly of the trans-envelope complexes. Transporters and MFPs exist in the inner membrane as dormant complexes. The activation of complexes is triggered by MFP binding to the outer membrane channel, which leads to a conformational change in the membrane proximal domain of MFP needed for stimulation of transporters. The activated MFP-transporter complex engages the outer membrane channel to expel substrates across the outer membrane. The recruitment of the channel is likely triggered by binding of effectors (substrates) to MFP or MFP-transporter complexes. This model together with recent structural and functional advances in the field of drug efflux provides a fairly detailed understanding of the mechanism of drug efflux across the two membranes.

## Transporters

Multidrug resistance or polyspecific transporters (MDRs) are present in all living systems. However, they are particularly abundant and diverse in bacteria and comprise 2–7% of the total bacterial protein content (Saier and Paulsen, 2001). Such putative MDRs are identified based on sequence similarity with experimentally confirmed transporters able to handle multiple substrates. Most of these substrates are hydrophobic or amphipathic molecules often containing weakly basic moieties. Other substrates are organic cations with a permanent charge distributed over a large hydrophobic surface (Hall et al., 1998; Zgurskaya and Nikaido, 2000b).

Functional studies and subsequent phylogenetic analysis demonstrated that bacterial MDR transporters can be organized into several evolutionary distinct protein families that significantly differ in bioenergetics, structure and transport mechanism (Saier and Paulsen, 2001). Most of MDRs are found in three large and diverse superfamilies: ABC (ATP-binding Cassette) (Higgins and Linton, 2004), MF (Major Facilitator) (Saier et al., 1999) and RND (Resistance-Nodulation-Cell Division) (Tseng et al., 1999). In addition, some MDRs form a core of smaller superfamilies: SMR (Small Multidrug Resistance) family [now part of the DMT (Drug/metabolite Transporter) superfamily] (Chung and Saier, 2001) and MATE (Multidrug and Toxic Extrusion) family [recently joined the MOP (Multidrug/Oligosaccharidyl-lipid/Polysaccharide) superfamily] (Hvorup et al., 2003). Just recently, a new family of transporters involved in efflux of cyclohexidine has been identified in *Acinetobacter* spp. (Hassan et al., 2013).

ABC MDRs (as all other members of this superfamily) are primary active transporters which couple substrate translocation with binding and hydrolysis of ATP. MDRs in all the other superfamilies are secondary transporters which utilize electro-chemical gradients of ions (most frequently protons but sometimes sodium) to transport their diverse substrates. Both primary and secondary transporters are ubiquitous in bacteria, however their relative presence seems to correlate with energy generation: fermentative bacteria tend to rely more on the primary transporters while genomes of aerobic bacteria contain somewhat more secondary transporters (Paulsen et al., 1998, 2000).

### Efflux across cytoplasmic membranes

At least three steps are common to various transporters during transport across cytoplasmic membranes: (i) binding of a substrate on the cytoplasmic or periplasmic side of the membrane, (ii) conformational changes in a transporter leading to re-orientation of the binding site to the other side of the membrane, and (iii) the release of the substrate. The conformational change leading to reorientation of substrate binding sites and relaxation of a transporter are usually energy-dependent steps, which is provided by either ATP hydrolysis (ABC pumps) or by a proton motive force (PMF) or sodium motive force. The basic mechanism of energization of transporters by ATP and PMF are well understood on examples of ABC and MF transporters (Davidson et al., 2008; Fluman and Bibi, 2009) but require further analyses in other transporters such as those belonging to the RND superfamily (Eicher et al., 2014).

The directionality of the transport is defined by binding affinity on either side of the membrane and the transport reaction is thought to be reversible at least in the case of PMF-dependent pumps (Smirnova et al., 2011; Fluman et al., 2012). However, RND pumps seem to be uni-directional with some of them transporting substrates only across the outer membrane (Nikaido and Pages, 2012). In these transporters, the substrate binding site is located in the periplasmic domain and is separated from the proton translocation pathway. Other RND pumps, such as the metal efflux pump CusBAC from *E. coli*, transport substrates across both the cytoplasmic and the outer membranes. This transporter comprises a network of methionine and charged residues traversing the transmembrane and periplasmic domains and involved in metal binding and transport (Su et al., 2011, 2012).

Transporters belonging to the same family of proteins are capable of efflux either alone or in complexes with accessory proteins. Mechanisms of transporters that function in complexes with other proteins are likely to be very similar to single-component ones. Studies of various transporters suggested that accessory proteins located in the periplasm and in the outer membrane enable high affinities toward substrates and also couple transport reactions separated in two different membranes.

### Efflux across outer membranes

Efflux is most effective when working in cooperation with other resistance mechanisms. Reduced uptake across the outer membrane of Gram-negative bacteria, which is a significant permeability barrier for both hydrophilic and hydrophobic compounds, constitutes such a mechanism (Sen et al., 1988; Sanchez et al., 1997). To take advantage of the reduced uptake, some Gram-negative MDR pumps extrude their substrates across the outer membrane directly into the external medium.

Permeability constants of amphiphilic antibiotics across cytoplasmic and outer membranes often differ by several orders of magnitude. Therefore, efflux is most effective when antibiotics are transported across the outer membrane back into the external medium. Among various MDRs, some transporters belonging to RND, ABC, and MF superfamilies utilize energy conserved in ATP or in the PMF of the cytoplasmic membrane to transport drugs across the outer membrane, which is energy deficient. This transduction of energy is possible because of the association between the transporters and two types of accessory proteins: the periplasmic Membrane Fusion Proteins (MFP) and the outer membrane channels (OMFs) (Paulsen et al., 1997).

Importantly, the trans-envelope efflux of antibiotics contributes to resistance in clinical settings. In clinical *Escherichia coli* and *Klebsiella pneumoniae* isolates, fluoroquinolone resistance is linked to overproduction of AcrAB efflux pump, and multiple efflux pumps, including MexAB-OprM, confer antibiotic resistance in *Pseudomonas aeruginosa* (Ziha-Zarifi et al., 1999; Padilla et al., 2010; Swick et al., 2011). Also in *P. aeruginosa*, elevated expression of MexXY is the major cause of aminoglycoside resistance in the absence of modifying enzymes (Poole and Srikumar, 2001). The unquestionably significant impact of multidrug efflux pumps on the effectiveness of antibiotics in clinical settings makes them attractive targets for inhibition (Lomovskaya et al., 2008a,b). This review is focused on the mechanism of coupling drug transport reactions in the cytoplasmic and outer membranes. This critical step in drug efflux could be targeted for development of effective inhibitors of efflux pumps.

## Outer membrane channels

The outer membrane of Gram-negative bacteria is an asymmetric lipid bilayer, which significantly slows down diffusion of drugs and bile salts into the cell. Although the inner leaflet of the outer membrane is composed of glycerophospholipids, the major components of cytoplasmic membranes, the outer leaflet is composed of lipopolysaccharides (LPS) which are largely responsible for the low permeability characteristic (Tommassen, 2010; Gessmann et al., 2014). Of the five existing families of Gram-negative outer membrane channels (Hagan et al., 2011), only proteins belonging to the Outer Membrane Factor (OMF) family associate with periplasmic MFPs and active transporters to drive the extrusion of ions, drugs, peptides and other substrates across the outer membrane (Yen et al., 2002). During transport, OMFs are believed to transition between the “open” and “closed” conformational states. In the resting state, the channel is in the “closed” conformation and is not permeable to most of its substrates. Induction of the conformational changes necessary to “open” the periplasmic aperture of OMFs is credited to the MFPs, which also stabilize the trans-envelope protein complexes and enable export of various substrates through OMFs (Matias et al., 2003; Lobedanz et al., 2007; Tikhonova et al., 2009).

### OMF structure

**Figure 1:**
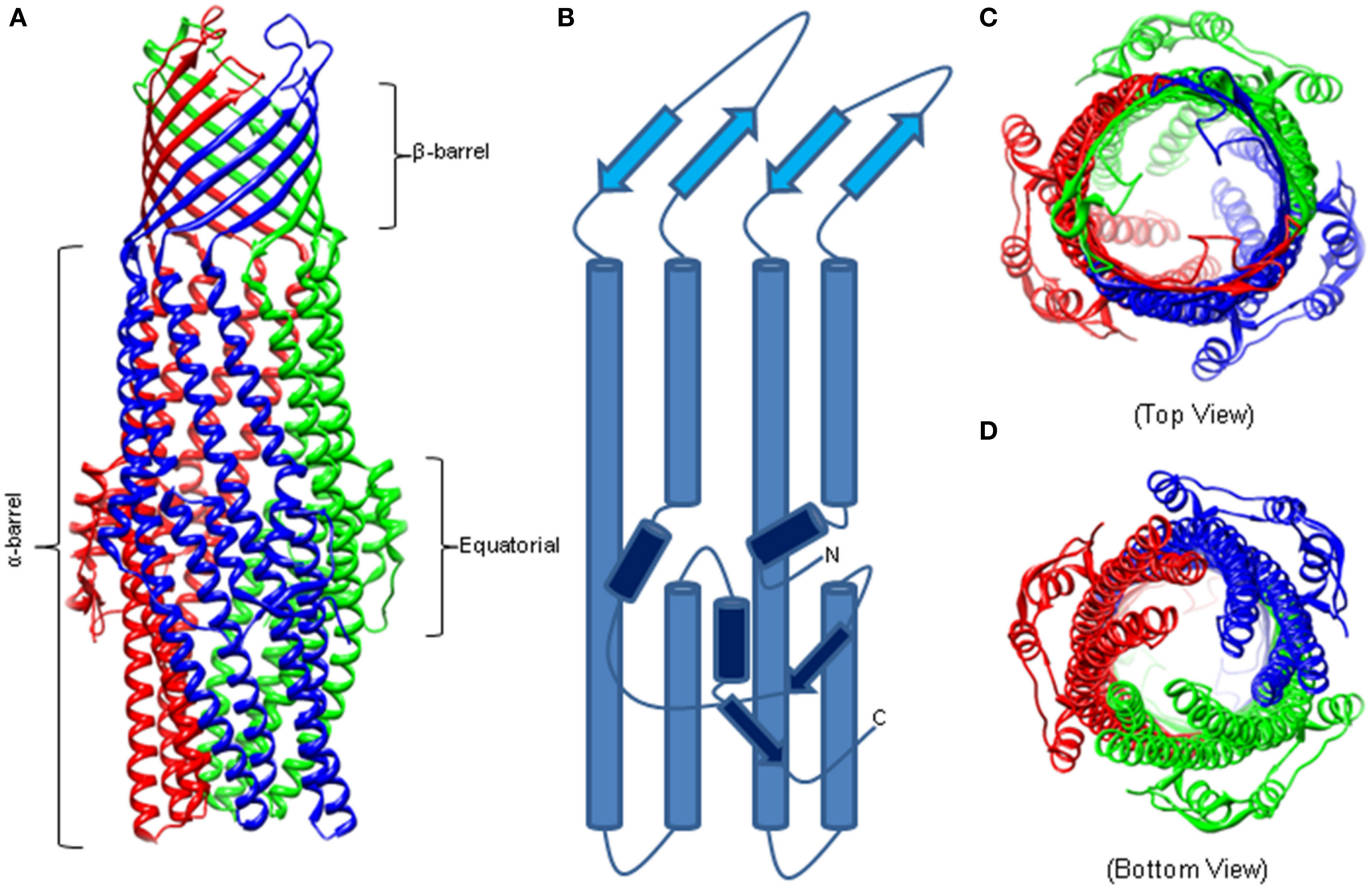
**The Structure of Homotrimeric TolC. (A)** Crystal structure of TolC (PDB code—1EK9). The individual protomers are colored. The β-barrel and extracellular loops are 40 Å long while the α-barrel is 140 Å deep into the periplasm. **(B)** A trace of an individual TolC protomer. The β-barrel domain is in light blue, the α-helical barrel in blue, and the equatorial domain in dark blue. **(C)** Crystal structure highlighting the open cavity of the TolC channel. **(D)** The “closed” resting state of the TolC periplasmic aperture.

Similar to other outer membrane proteins, OMFs have a signature β-barrel domain but these proteins also have unique features befitting their functions in the trans-envelope transport. The first OMF purified and characterized was the homotrimeric TolC from *E. coli* that functions with various drug efflux pumps of RND (e.g., AcrAB), ABC (e.g., MacAB) and MF (e.g., EmrAB) superfamilies (Figure 1) (Koronakis et al., 2000). TolC is a 12 β-strand barrel, which is 30 Å wide and contains six asymmetric loops exposed to the cell surface. The β-barrel extends into the periplasm as an α-barrel of 140 Å long. The α-barrel is formed by 12 α-helices—six continuous and six discontinuous—that share similar sequences and inter-helical interactions, yet one pair of helices is inclined by −20° with respect to the molecular threefold axis. The introduced curvature yields a “closed” aperture of the periplasmic end of TolC. The α-barrel domain is belted by an α/β structure that comprises the equatorial domain.

TolC homologs crystallized since then, MtrE, VceC, OprM, and CusC, conserve closely the α- and β-barrel architecture (Akama et al., 2004a; Federici et al., 2005; Kulathila et al., 2011; Lei et al., 2014). The equatorial domain is also retained but with significant variation, particularly in the C terminus (Federici et al., 2005; Kulathila et al., 2011). Highlighting the important role of the equatorial domain, mutations in the N and C termini attenuate the function of TolC (Yamanaka et al., 2002, 2004; Bokma et al., 2006; Krishnamoorthy et al., 2013).

Most of the available structures of OMFs, including TolC, OprM and CusC are closed at one or both sides. Several structures of the TolC variants, in which the opening of the TolC exit duct was forced by removal of key interactions, have been reported (Andersen et al., 2002; Bavro et al., 2008; Pei et al., 2011). A network of inter- and intra-protomer bonds that constrains the inner and outer coiled coils in their closed conformation is centered at four key residues: Thr152, Asp153, Tyr362, and Arg367. Disruption of key hydrogen bonds and salt bridges involving these residues allowed movement of the inner coiled coils and widened the periplasmic pore (Andersen et al., 2002). Structural characterization of these TolC mutants presented sequential snapshots of both symmetrical and asymmetrical openings of the entrance aperture, revealing that relaxation of the constraining network allows both a twist and dilation at the entrance (Bavro et al., 2008; Pei et al., 2011). These studies also suggested that interactions with transporters and periplasmic proteins are likely to contribute significantly to opening of the channel.

**Figure 2:**
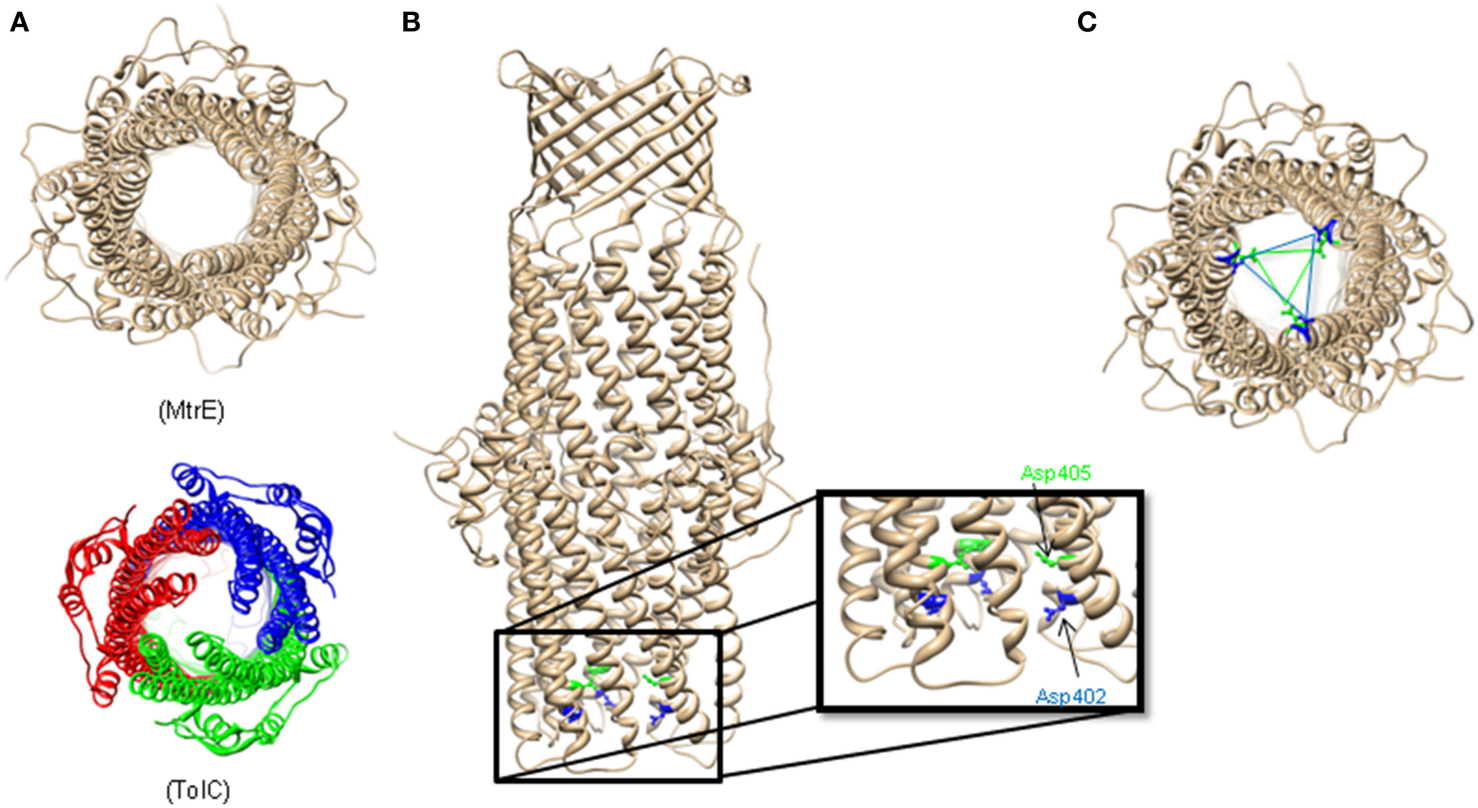
**The Structure of *Neisseria gonorrhoeae* MtrE**. **(A)** Bottom view of the open conformational state of MtrE (generated by SWISS-MODEL) compared to TolC (bottom). The narrowest region of MtrE tunnel is ~12 Å. **(B)** Acidic residues of the α-helical barrel of MtrE contribute to substrate selectivity. **(C)** A unique feature at the MtrE periplasmic opening—two negatively charged concentric rings composed by Asp402 (blue) and Asp405 (green).

Surprisingly, the crystal structure of MtrE showed that this channel is open all the way from the outer membrane surface and down to the tip of the α-helical periplasmic domain (Figure 2) (Lei et al., 2014). The widest section of the channel is located at the external surface of the outer membrane, with the internal diameter of ~22 Å. Hence, MtrE could be a highly dynamic channel spontaneously transitioning between the closed and open states. Like TolC and other OMFs, MtrE also contains an aspartate ring at its periplasmic entrance. Each protomer of MtrE contributes Asp402 and Asp405 to form two concentric circles of negative charges in the inner cavity of MtrE, which could contribute to the selectivity of the channel (Figure 2).

### Proposed mechanism

The structural and functional studies suggested that opening of the channel could be achieved by interrupting inter- and intramolecular interactions that stabilize the resting state. Interactions with both a transporter and a MFP were considered as driving forces for OMF transition into the open state.

The *E. coli* K-12 periplasm is estimated to range ~18–23 nm (Matias et al., 2003). In case of RND-containing complexes, the periplasmic portion of the AcrB transporter is large enough to reach halfway across the periplasm and contact TolC directly (Murakami et al., 2002). If AcrB and TolC structures are docked in a tip-to-tip manner the complex spans ~17 nm of the periplasm and the drug exit funnel of AcrB is sealed by the periplasmic duct of TolC preventing the escape of substrates into periplasm (Murakami et al., 2002; Symmons et al., 2009). This model has found a support at both the genetic and biochemical levels. Binding experiments showed that the transporter-channel AcrB-TolC pair forms a highly dynamic complex, even in the absence of MFP AcrA (Tikhonova et al., 2011). An “open” TolC variant containing Tyr362 to Phe and Arg367 to Glu substitutions (TolC^YFRE^) showed significantly lower affinity to AcrB (Tikhonova et al., 2011), suggesting that opening of the channel could lead to destabilization of interactions, disengagement of the complex and channel closing. The proposed AcrB-TolC interface is formed by AcrB β-hairpins extending from its TolC-docking domain. Genetic studies showed that these hairpins play an important role in the functional assembly of the pump complex by stabilizing interactions with TolC (Weeks et al., 2014). However such a limited interface could initiate the opening of the channel but is not sufficient to release interactions that keep the channel in its closed state (Weeks et al., 2010). Interestingly, in the assembled tri-partite AcrAB-TolC complex, AcrB and TolC do not contact each other (Du et al., 2014). Also, transporters belonging to ABC and MF superfamilies of proteins lack the large periplasmic domains of AcrB and do not interact with OMF directly (Lu and Zgurskaya, 2013). Hence the transporter-channel interface, if formed, is not a requirement for assembling a functional complex. This function is assigned to MFPs that play a critical role in coupling transport reactions separated in two different membranes.

## Coupling of two membranes: periplasmic membrane fusion proteins

MFPs play an important functional role in trans-envelope drug efflux: (i) by stimulating the efflux activities of transporters and (ii) by engaging OMFs and opening them for substrates to be expelled from the cell (Zgurskaya et al., 2009). *In vitro* reconstitution studies showed that the stimulating activities of MFP are a common feature of transporters belonging to various protein families. *E. coli* AcrA, that functions with the RND pump AcrB, and MacA, a subunit of the ABC-type MacB transporter, both stimulate activities of their cognate transporters located in the inner membrane (Zgurskaya and Nikaido, 1999b; Tikhonova et al., 2007). On the other hand, MFPs mediate and stabilize the functional interactions between transporters and OMFs and in doing so, presumably transmit energy from the energized transporter to the closed OMF in order to open the channel and allow the diffusion of substrates through the OMF into the extracellular medium. It is thought that this transfer of energy is mediated by conformational energy and movement of the MFPs.

### Structure of MFPs

**Figure 3:**
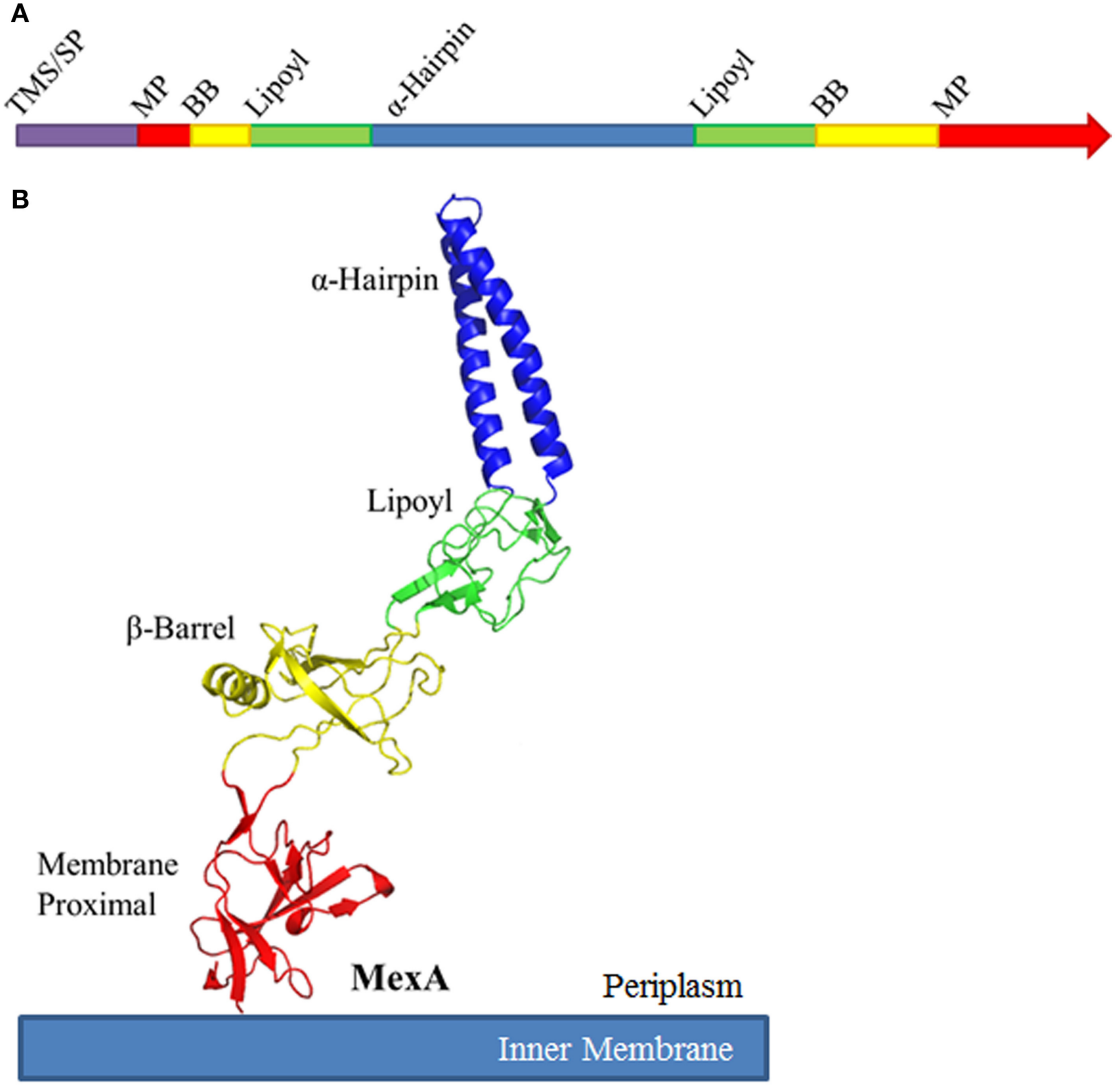
**Schematic organization of Membrane Fusion Proteins**. **(A)** Typical membrane fusion proteins (MFPs) contain four domains which are broken and distributed between the N- and C-halves of the protein. As MFPs are periplasmic proteins, they contain an N-terminal transmembrane segment (TMS) or signal peptide (SP). The N-terminal half of the protein encodes for a small portion of the membrane proximal (MP) and β-barrel domains (BB), half of the lipoyl domain and one of the α-helices of the α-helical domain. The C-terminal half encodes for the second α-helix, the second half of the lipoyl domain and the majority of the BB and MP domains. **(B)** When folded, MFPs form an elongated structure with four unique domains. From the inner membrane, the domains are organized as MP, BB, lipoyl and α-helical. The domains are separated by short flexible linkers. Due to the flexibility of these linker regions, MFPs are capable of dramatic variation in structure, likely to allow for conformational changes which transmit energy from the IMP to the OMF. The MP, BB, and lipoyl domains are primarily comprised of β-sheets, while the α-helical domain is typically a single pair of anti-parallel α-helices connected by a single turn.

While amino acid conservation among MFPs is relatively low, their structures are conserved. Typical MFPs are highly elongated proteins, and adopt a linear conformation that extends from the outer leaflet of the inner membrane deep into the periplasm to meet the OMF (Zgurskaya and Nikaido, 1999a; Mikolosko et al., 2006). This linear structure comprises up into four characteristic domains. Starting at the inner membrane, the domains are the membrane proximal (MP), β-barrel, lipoyl, and α-helical hairpin domains (Figure 3). While these domains are arranged in a linear manner, the N-terminal and C-terminal halves of the protein fold in upon each other to complete the structure.

The MP, β-barrel and the lipoyl domains are mostly β-sheet structures. The α-hairpin domain, unlike the other three domains, consists of two anti-parallel coiled-coils separated by a single turn. The domains are linked by flexible unstructured regions which give the overall protein its conformational flexibility and allow for a high degree of movement believed to be important for functional interactions with transporters and OMFs.

**Figure 4:**
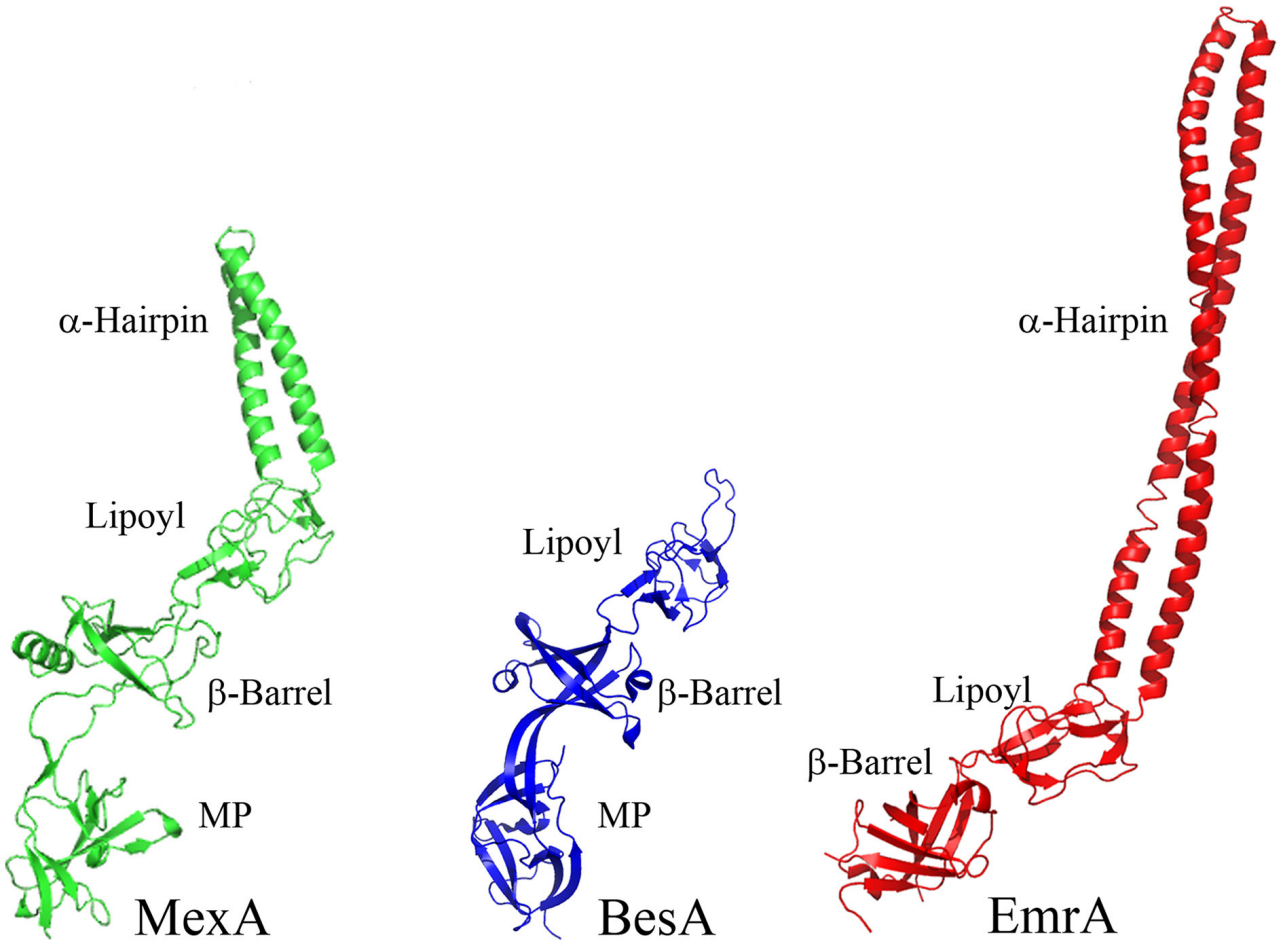
**Structures of divergent MFPs**. Crystal structures of MexA, BesA, and EmrA are shown to show structural variation of MFPs (PDB accession numbers 2V4D, 4KKS, and 4TKO, respectively). The typical MFP is exemplified by the structure of MexA, which contains all four of the typical domains. BesA is the MFP of the BesABC RND efflux complex of *B. burgdorferi*. The OMF, BesC, does not contain both of the ionic gates which keep the periplasmic aperture in a closed position. Due to this lack of additional gating, it is thought that BesA no longer needs the α-helices because of the weakened gating of the aperture. Conversely, EmrA interacts with the MFS transporter EmrB. EmrB contains little to no periplasmic domain for EmrA to interact with. It is thought that EmrA interacts with EmrB primarily through the N-terminal transmembrane segment which anchors EmrA to the membrane and not through the MP domain. Due to the loss of the MP domain in EmrA, the α-helical domain has been extended. This extended α-helical domain allows for EmrAB to interact properly with TolC so as to allow efflux of substrates into the extracellular space.

The α-helical hairpins oligomerize and assemble a funnel-like structure that interacts with an OMF. The MP, β-barrel, and possibly the lipoyl domain are responsible for interactions with the cognate transporter. In addition, some MFPs, such as MacA, contain N-terminal transmembrane domains (TMD) that interact with transporters within the cytoplasmic membrane (Tikhonova et al., 2007). However, in many other MFPs this TMD is replaced by a lipid modification that anchors the protein into the membrane. As the different families of transporters vary in sizes of domains exposed to the periplasm, MFPs and their domains which interact with specific transporters also vary. For example, MF transporters such as *E. coli* EmrB are thought to have little to no periplasmic extrusions (Figure 4). Accordingly, its cognate MFP, EmrA, lacks a MP domain and contains a very long α-hairpin sufficient to span the periplasm and bind TolC (Hinchliffe et al., 2014). On the other side of the MFP diversity spectrum is BesA, the MFP of *Borrelia burgdorferi* BesABC complex, which lacks an α-hairpin domain all together (Greene et al., 2013). The exact reasons for the lack of the α-hairpin remains unclear, but it could be due to the fact that OMF, BesC, lacks the aspartate ring in the periplasmic duct of the channel suggesting that MFP binding to the periplasmic tip of BesA could be sufficient to trigger opening of the channel (Bunikis et al., 2008).

### Interactions between MFPs and other complex components

#### Oligomerization of MFPs

MFPs oligomerize independently of interactions with their cognate IMPs and OMFs. The first evidence of MFP oligomerization came from *in vivo* cross-linking studies of AcrA and HlyD (Thanabalu et al., 1998; Zgurskaya and Nikaido, 2000a). More recently, structural studies showed that in crystals MFPs such as *P. aeruginosa* MexA and *E. coli* MacA, and AcrA, all have repeating dimer pairs (Akama et al., 2004b; Mikolosko et al., 2006; Yum et al., 2009). AcrA is a dimer of dimers, MacA forms a hexameric funnel-like structure, whereas MexA has sets of dimers aligned with one another creating a sheet, which wraps upon itself in an asymmetric fashion. These structures suggested that the MFPs could function as dimers, but the strongest evidence for a dimer as a functional unit of MFPs was collected by biochemical and genetic studies (Mima et al., 2007; Tikhonova et al., 2009, 2011; Xu et al., 2011a).

For MFPs which interact with RND transporters (e.g., MexA and AcrA), the MFP oligomerization was treated more as an artifact of crystallization, partly due to the fact that hydrodynamic studies and the size exclusion chromatography studies showed only monomers in solution (Zgurskaya and Nikaido, 1999a; Tikhonova et al., 2009). However, kinetic studies established that MFPs that function with transporters belonging to different protein families all oligomerize, albeit to a different degree (Tikhonova et al., 2009). EmrA and MacA, MFPs functioning with MFS and ABC transporters, respectively, form more stable oligomers and with a higher affinity than AcrA. This result suggested that interactions with the periplasmic domains of AcrB stabilize the oligomeric state of AcrA. In contrast, MFPs that function with transporters lacking large periplasmic domains have a strong propensity to self-oligomerization. These studies suggested that oligomerization of MFPs is needed to seal the gap between their cognate transporters and TolC.

Further *in vivo* (construction of genetic fusions, Xu et al., 2011a) and *in vitro* (binding affinities of stabilized dimers Tikhonova et al., 2011) studies showed that the functional unit of MFPs is a dimer, which assembles into a trimer of dimers when in the complex. Perhaps the most natural evidence of MFP dimers, as functional units, is the *P. aeruginosa* triclosan-specific TriABC-OpmH complex. Mima et al. (2007) identified this complex through a screen for increasing triclosan resistance and discovered that TriA and TriB are unique MFPs which are both required for functionality of the complex.

#### Interactions between MFPs and transporters

Physical interactions between MFPs and transporters remain a subject of intense investigation using such approaches as crystallography, mutagenesis, surface plasmon resonance, iso-thermal calorimetry, chemical crosslinking, co-purification, and chimeric protein studies (Weeks et al., 2010, 2014; Su et al., 2011; Tikhonova et al., 2011; Xu et al., 2011a,b). Symmons et al. (2009) proposed the first model of AcrAB-TolC complex based on crystal structures of individual components, *in vivo* chemical crosslinking and molecular docking analyses. In this model, the AcrA molecule occupies the AcrB surface between the PN2 and PC2 subdomains, extending up along the DN subdomain. The MP, β-barrel, and lipoyl domains of AcrA are modeled to interface with AcrB. Interestingly the PC1 and PC2 cleft of AcrB has been proposed to be one of the main entrance tunnels which substrates use to enter into the drug binding pocket (Pos, 2009). This further suggested that MFPs could stimulate activities of their cognate transporters by participating in drug binding.

In the co-crystal structure of the metal efflux pump CusBA, the two CusB protomers are positioned substantially higher on the surface of the CusA transporter than in the cross-linking based model of AcrAB described above (Symmons et al., 2009; Su et al., 2011). Here, the MP and β-barrel domains of CusB interface primarily with the DN and DC subdomains of CusA and only make limited contacts with the top portions of the PN2, PC1, and PC2 subdomains. The authors were able to define two different types of MFP-transporter interactions. The first CusB molecule primarily engages CusA through an extensive network of salt bridges. Conversely, the second CusB molecule interacts with CusA primarily through a network of hydrogen bonding. In addition, the two CusB are staggered: one of the two molecules sits slightly higher on the surface of CusA. In this arrangement, the MP domain of CusB molecule 1 fits into a pocket formed between the MP and β-barrel domains of molecule 2. When compared with the cross-linking based model of AcrAB, CusB molecule 1 is positioned similar to AcrA with the exception that the lipoyl domain of CusB does not interface with the transporter and its MP occupies the position of β-barrel domain of AcrA. This upward shift also tilts the MFPs and gives them a different overall structure; CusB has a sickle shape as observed in crystal structures of other MFPs including MacA, AcrA, and MexA. The recently reported cryo-EM-based structure of the complete AcrAB-TolC complex shows similar positioning of AcrA on AcrB transporter (Du et al., 2014). However, only lipoyl domains of AcrA contribute to AcrA oliomerization and there are no contacts between the MP domains of AcrA.

In various models of MFP-transporter complexes, at least two domains of MFP directly contact a transporter: the MP domain and the β-barrel. Functional importance of these domains in various MFPs has been supported by genetic and biochemical studies (Elkins and Nikaido, 2003; Tikhonova et al., 2007; Ge et al., 2009; Modali and Zgurskaya, 2011). Mutagenesis of the C-terminal domains of MacA and AcrA identified conserved glycine residues important for the functional interactions between these MFPs and the cognate components of the complexes. A substitution of Gly353 with Cys leads to inactivation of MacA, and prevents stimulation of the MacB ATPase activity, but not physical interactions with MacB or TolC (Modali and Zgurskaya, 2011). An analogous alteration in AcrA, Gly363 to Cys, prevented functional, but not physical interaction with AcrB and TolC (Ge et al., 2009). Limited proteolysis studies both *in vivo* and *in vitro* showed that these substitutions affect conformations of the MP domains of AcrA and MacA. Although EmrA does not have a MP domain, its C-terminal signature residues of the MFP family of proteins are incorporated into the β-barrel (Zgurskaya et al., 2009; Hinchliffe et al., 2014).

#### Conformational changes and effectors

Early hydrodynamic and EPR studies pointed to significant structural flexibility of MFPs (Zgurskaya and Nikaido, 1999a; Ip et al., 2003). In the presence of magnesium ions, the highly asymmetric shape of AcrA was found to be more compact, whereas mildly acidic pH restrained significantly conformational dynamics of this protein. The X-ray structure of AcrA suggested that these conformational changes could involve the α-hairpin, which undergoes an ~15° rotation between the two most dissimilar molecules (Mikolosko et al., 2006). In addition, the refined full structure of MexA showed that the MP domain is also mobile and that in one of the MexA protomers this domain is twisted by 85° clockwise relative to the β-barrel domain (Symmons et al., 2009). Additionally, when the lipoyl domains of MexA were superimposed, the α-hairpins exhibited ~35° of rotation while the β-barrel and MP domains underwent an additional ~25° of rotation. Molecular dynamics simulations of MexA and AcrA supported the high inter-domain flexibility of MFPs (Vaccaro et al., 2006; Wang et al., 2012). *In vivo* proteolysis studies however, pointed onto the MP domains of AcrA and MacA that undergo significant conformational changes and that these changes are detected only when all three components of complexes are present and functional (Ge et al., 2009; Modali and Zgurskaya, 2011; Lu and Zgurskaya, 2012).

The protonation/deprotonation of His285 was proposed to underlie the pH-regulated conformational dynamics of AcrA by disturbing the local hydrogen bond interactions (Wang et al., 2012). Interestingly, in a heavy metal MFP ZneB, the same His residue is a part of the metal-binding site (De Angelis et al., 2010). In MacA, the patch of positively charged residues located at the interface between β-barrel and the MP domains are important for both binding to core LPS and functionality of the pump, suggesting that LPS could be a substrate of MacAB-TolC (Lu and Zgurskaya, 2013). In addition, kinetic experiments showed that AcrA and other MFPs show differential binding affinities to OMF and transporters depending on pH (Tikhonova et al., 2009, 2011). Hence, the changes of pH in the periplasm accompanying the drug efflux or binding of substrates/effectors could act as a signal to trigger the action of MFPs, which undergo reversible conformational rearrangement.

Several genetic screens with defective complexes were carried out to identify gain-of-function suppressor mutations in MFPs. Surprisingly, most of such suppressors were mapped to the β–barrel domains of MFPs, which do not contact OMFs directly and suppress the defects in complexes through the contact with transporter and long range conformational changes in the protein. Suppressors of defects in MexB transporter mapped to the β-barrel domain of MexA were found to increase the stability of MexA binding to MexB (Nehme and Poole, 2007). An analogous study of an assembly defective TolC mutant also gave rise to gain-of-function suppressor mutations within the β-barrel domain of AcrA (Gerken and Misra, 2004). One such suppressor of the defective TolC mutant was AcrA_L222Q_, which is itself labile and subject to degradation if not in a functional complex with AcrB and TolC (Weeks et al., 2010). It is thought that this mutant protein is in a conformation which AcrA assumes transiently during typical complex assembly and drug efflux; however, when locked into this conformation, the protein becomes highly sensitized to proteolytic degradation by the periplasmic protease DegP. Other AcrA β-barrel mutants suppressed a TolC mutant defective in functional interactions with AcrA and AcrB presumably by controlling the opening of TolC channel (Weeks et al., 2010), or enabled functioning of a hybrid AcrA-MexB-TolC efflux complex (Krishnamoorthy et al., 2008).

## Proposed mechanism of trans-envelope drug efflux

**Figure 5:**
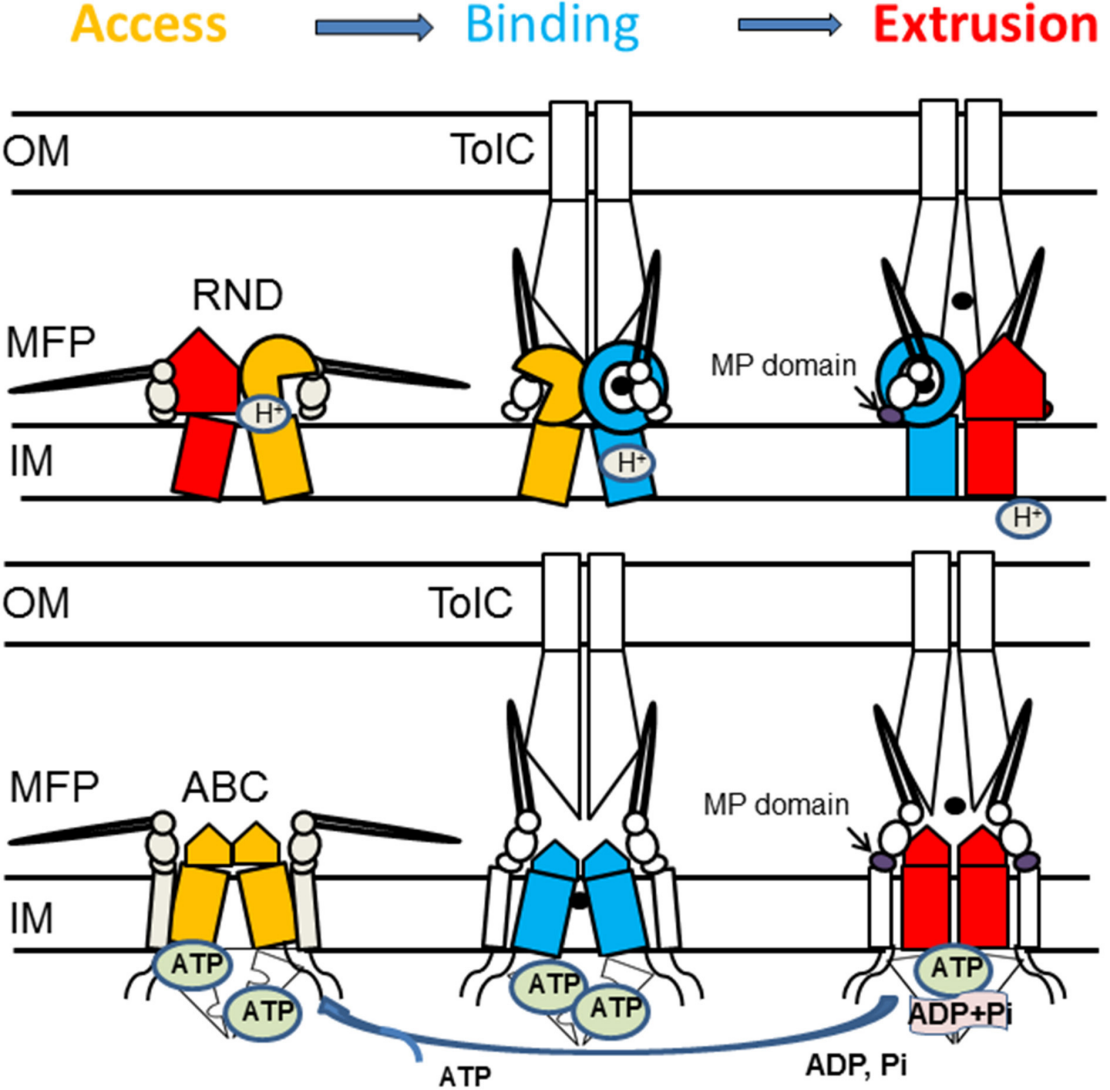
**Proposed mechanism of assembly and trans-envelope transport by tri-partite efflux pumps**. Despite different structures and mechanisms, RND and ABC transporters cycle through three states that promote access, binding and extrusion of substrates. In RND transporters, each protomer cycles through the three conformations. The protomer in the substrate bound state (blue) is likely to be protonated and its transition into the extrusion state (red) is coupled to influx of protons. Dimeric ABC transporters are saturated with ATP, whereas binding of the substrate leads to transition into a semi-closed (middle panel) conformation. MFPs stabilize the substrate-bound state of transporters (blue) and drive transporters into the closed state needed for ATP hydrolysis. Association with TolC drives conformational changes in the MFP needed for stimulation of transporters.

Studies discussed above suggest that efflux complexes assembled with transporters belonging to various protein families utilize the same mechanism of transport across the outer membrane and that MFPs play an important role in the functional communication between transporters and OMF. Although molecular details of how ABC, MF, and RND transporters bind their substrates and transport them to the OMFs differ significantly between members of different families, all these transporters cycle through three states that promote access, binding and extrusion of substrates (Figure 5). The function of MFPs is to couple these transitions in transporter to opening of OMFs and transport of substrates across the outer membrane. Most of the investigations were done on either RND or ABC efflux complexes but it is likely that the same conclusions apply to MF complexes. The major findings on the mechanism of MFPs in transport could be summarized in the following conclusions:

1. MFP-transporter interactions are sensitive to conformational changes in transporters. When MacB transporter was trapped in different conformations by mutations or by the presence of nucleotide analogs, the affinity of association between MFP MacA and MacB increased upon ATP binding but remained unchanged during ATP hydrolysis (Lu and Zgurskaya, 2012). Similarly, the interactions between the MFP AcrA and the RND transporter AcrB are most stable when AcrB is in its protonated state (Janganan et al., 2013). These results suggested that MFPs act on the pre-translocation “Binding” state of transporters (Figure 5).
2. Stimulation of transporters is coupled to a conformational change in the C-terminal domain of MFPs. Reconstitution studies showed that interactions between MFPs and transporters increase the rates of transport and energy consumption (Zgurskaya and Nikaido, 1999b; Tikhonova et al., 2007). Further studies of MacAB and AcrAB showed that the C-terminal domains of MFPs are critical for their ability to stimulate transporters (Ge et al., 2009; Modali and Zgurskaya, 2011). These domains of MFPs interface with periplasmic domains of transporters and undergo conformational changes needed for stimulation of transporters. Studies of MacAB-TolC suggested that MFPs stimulate ABC transporters by stabilizing the closed pre-ATP hydrolysis conformation (Modali and Zgurskaya, 2011). Assuming conservation of the mechanism, we propose that in the case of RND transporters, MFPs stabilize the binding-to-extrusion transition that precedes the transmembrane proton transfer (Figure 5).
3. Interaction with OMF is needed for conformational changes in MFPs. The *in vivo* proteolysis approach showed that *in vivo* conformational changes in MFPs occur only in the presence of TolC (Ge et al., 2009; Modali and Zgurskaya, 2011). The TolC-induced conformational changes in MFPs were further linked to stimulation of activities of transporters by MFPs and to stabilization of tri-partite complexes *in vitro*. Taken together these results support the model that *in vivo* MFPs exist in stable complexes with transporters and that association with OMF triggers the conformational changes in MFPs needed for stimulation of transporters (Figure 5).
4. Interactions between transporters and OMFs are dynamic. Current models postulate that OMF association with an MFP-transporter complex leads to opening of the channel (Weeks et al., 2010; Janganan et al., 2013). In the docked models of AcrAB-TolC, TolC is in the open conformation (Symmons et al., 2009; Du et al., 2014). The dilation of the TolC channel in the assembled complex is apparently driven by energy-dependent conformational changes in MFP-transporter complexes (Janganan et al., 2013). The open and closed conformers of TolC stabilized by mutations can be readily distinguished *in vivo* by drug susceptibility, proteolytic and thiol labeling profiles (Krishnamoorthy et al., 2013). However, the interactions with TolC are highly dynamic and the life-time of TolC-containing complexes is very short (Tikhonova et al., 2009, 2011). Thus, TolC binding to the inner membrane complexes is transient.

Based on the data described above, we propose that reaction cycles of transporters are tightly coupled to the assembly of the trans-envelope complexes. Transporters and MFPs exist in the inner membrane as inactive complexes (Figure 5). The activation of complexes is triggered by MFP binding to OMF, which leads to a conformational change in MFP needed for stimulation of transporters. The activated MFP-transporter complex engages an OMF to expel substrates across the outer membrane. The recruitment of an OMF is likely triggered by binding of effectors (substrates) to MFP or MFP-transporter complex. This possibility has been discussed before for the *P. aeruginosa* MexJK pump where experimental evidence strongly suggests that the MexJK complex recruits either OprM or OpmH in a substrate-dependent matter (Chuanchuen et al., 2005).

## Non-typical drug efflux transporters

The discussed above MFP-transporter complexes have a three-fold symmetry befitting the trimeric architecture of OMF. However, asymmetric assemblies like RND transporters MdtABC or TriABC are also broadly represented in bacterial genomes. The mechanism and the requirement for asymmetry in such complexes remain unclear.

MdtB and MdtC share only 50% sequence identity with each other. The expression of both MdtB and MdtC together with the MFP MdtA leads to a decreased drug susceptibility of *E. coli* to SDS, novobiocin, cloxacillin, and several bile salts (Baranova and Nikaido, 2002; Nagakubo et al., 2002; Kim et al., 2010; Kim and Nikaido, 2012). The expression of MdtAB does not yield a functional transporter, but expression of MdtAC resulted in partial activity: a drug resistant phenotype against bile salts. Co-purification experiments and protein fusion studies suggest that MdtBC associates as a B_2_C complex (Kim et al., 2010). Furthermore, there is convincing evidence that MdtB and MdtC fulfill distinctly different roles for the transporter. Site-directed mutagenesis studies have shown that a mutation in the proton relay network of MdtB abolishes functionality of the complex to a greater extent than corresponding mutations in MdtC (Kim et al., 2010). On the other hand, mutations in the proposed drug binding pocket and 3D docking studies on homology models indicate that substrates bind primarily to MdtC (Kim and Nikaido, 2012). The proton relay network in MdtB is thus thought to provide the energy required for a conformational change in MdtC resulting in removal of substrates (Mima et al., 2009). This arrangement differs from the current model of drug expulsion in homotrimeric RND transporters, in which each protomer is functionally identical. MuxABC-OpmB in *P. aeruginosa* similarly includes two RND components in a single operon MuxB and MuxC. In this system, both peptides are necessary in order to observe drug resistance against a range of antibiotics including novobiocin and carbenicillin (Mima et al., 2009; Yang et al., 2011).

In some complexes, the asymmetry arises through hetero-oligomerization of MFPs. TriABC-OpmH from *P. aeruginosa* requires two MFPs TriA and TriB that share 36% sequence identity with each other (Mima et al., 2007). TriABC-OpmH provides resistance to triclosan with both TriA and TriB required for efflux (Mima et al., 2007). TriA and TriB presumably form either a trimer of dimers or a hetero-oligomeric hexamer. Since both MFPs share relatively little sequence identity and each of them is required for activity, it is likely that they serve different functions for the transporter.

**Figure 6:**
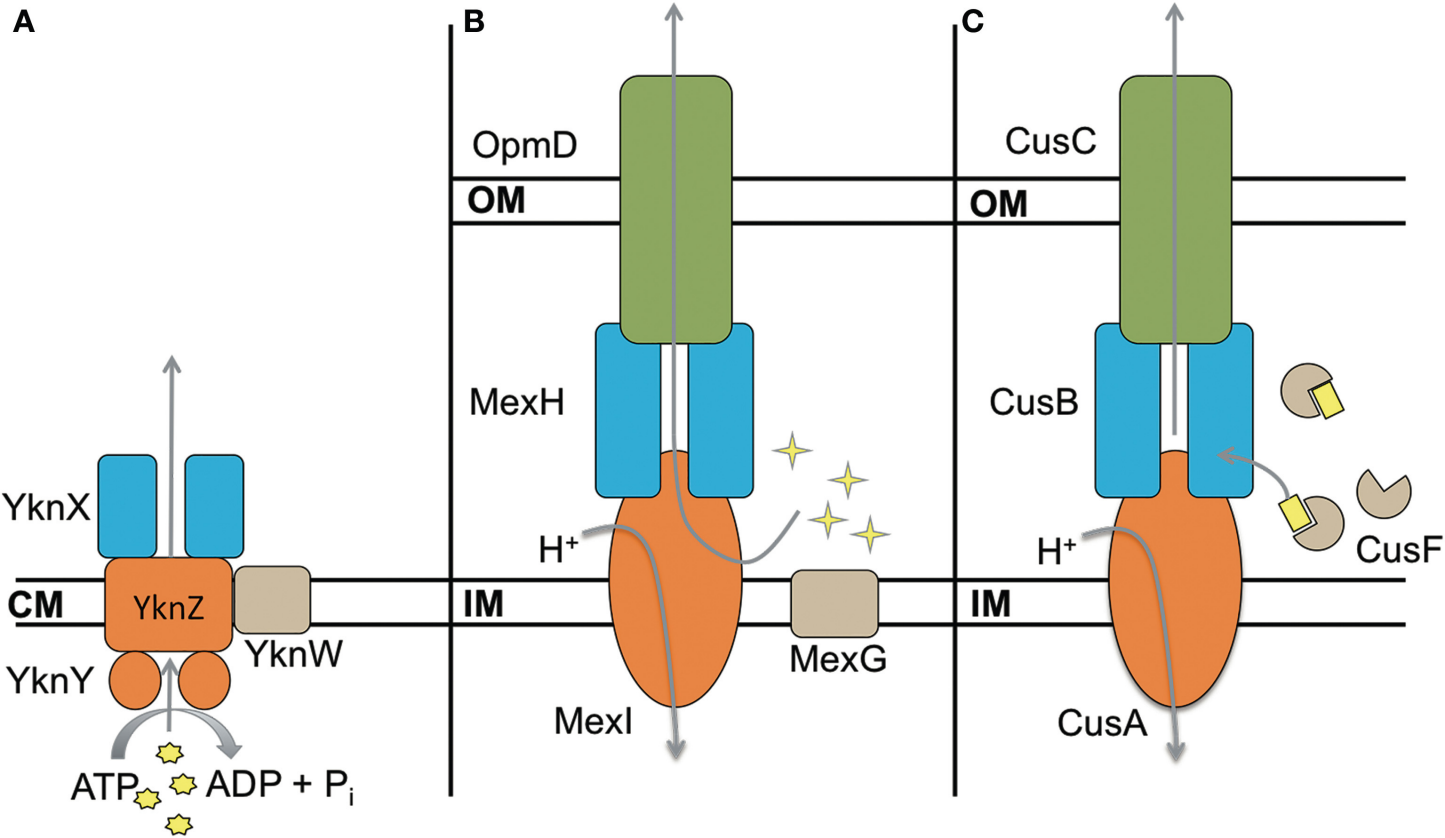
**Four component transporters**. **(A)** ATP type transporter YknWXYZ from *B. subtilis*. YknW is thought to associate with the YknXYZ complex and seems to be essential for the transporter (Yamada et al., 2012). **(B)** MexGHI-OpmD is an RND transporter in *P. aeruginosa* that is involved in virulence (Aendekerk et al., 2002, 2005). MexG is proposed to be located in the inner membrane and contributes to the functionality of MexHI-OpmD (Sekiya et al., 2003). **(C)** RND transporter CusBAC-CusF from *E. coli*. CusF is a metal chaperon that is thought to donate Cu(I) to the MFP CusB (Argüello et al., 2011; Mealman et al., 2011; Padilla-Benavides et al., 2014). Shapes in yellow represent the corresponding substrates for each transporter.

Some transporters include a fourth unique component in their operons. These proteins are typically not required for functionality of the complex, however their presence modifies the activity of the transporter. The best characterized efflux pumps with a fourth component are the ABC transporter YknWXYZ from *B. subtilis* (see below) (Yamada et al., 2012), MexGHI-OpmD from *P. aeruginosa* (Aendekerk et al., 2002) and CusF-CusBAC from *E. coli* (Kim et al., 2011) (Figure 6). In addition, there are small ~50 aa long peptides like AcrZ from *E. coli* that binds to AcrAB-TolC and modifies its substrate specificity (Hobbs et al., 2012).

MexGHI-OpmD is required for virulence of *P. aeruginosa* and its overproduction decreases susceptibilities to norfloxacin, ethidium bromide, acriflavine, and rhodamine 6G (Aendekerk et al., 2002, 2005; Sekiya et al., 2003). The complex is thought to be involved in the translocation of quorum sensing molecules that upregulate the transcription of virulence factors. MexH, MexI and OpmD are MFP, RND, and OMF respectively, whereas MexG is a small 16.2 kDa protein of unknown function. According to sequence analysis it is proposed to be a membrane protein comprising four transmembrane α-helices. MexHI-OpmD is functional without MexG but the latter is required for full functionality of the complex (Sekiya et al., 2003). Homologs of MexG exist in other Gram-negative bacteria that are also encoded in operons with RND transporters, suggesting that the requirement for the fourth component is conserved. More work is needed to elucidate the role of MexG homologs.

The existence of asymmetric and four-component complexes implies that current models of the MFP-dependent transport may be incomplete. It is possible that the asymmetry and additional components split functional roles of subunits and promote one of the transition states. What step(s) in the transport could be enhanced by additional subunits? In case of MFPs, this could be the separation of roles in the stimulation of a transporter and the recruitment/opening of OMF. In transporters, this could be an added substrate specificity or a separation of energy transduction and substrate translocation as suggested for MdtBC (Nagakubo et al., 2002; Kim et al., 2010). It is also possible that the hetero-oligomerization of transporters and MFPs as well as additional components might serve similar roles in the transport cycle such as modulation of transporter affinities to substrates and/or MFPs and OMFs. In this way, asymmetry might increase the specificity of a MFP-transporter complex to an OMF, which in turn could be a way to control the activity of the transporter.

## MFP-dependent transporters of gram-positive bacteria

Although MFPs are ubiquitous in efflux complexes that function in the context of two membranes, these proteins are also present in Gram-positive bacteria, which typically lack an outer membrane. Overall, the structures of cell envelopes of Gram-positive bacteria are poorly characterized, and while Gram-positive and Gram-negative cell envelopes share some characteristics, they are chemically and compositionally distinct. MFPs are required for functions of many transporters in Gram-positive bacteria that are involved in drug resistance, membrane biogenesis, and protein secretion (Butcher and Helmann, 2006; Yamada et al., 2012; Freudl, 2013).

Sequence homology and modeling indicate that MFPs from Gram-positive bacteria share the same general architecture as Gram-negative MFPs (Figure 3). The conservation of the α-hairpin region in Gram-positive MFPs is puzzling, as the region is thought to be the major contributor toward interactions between the MFP and OMF in Gram-negative transporters. The best characterized complex is *B. subtilis* YknWXYZ known to provide resistance against the endogenously-produced toxin SdpC, which lyses surrounding cells, freeing nutrients and allowing the cell to delay sporulation (Butcher and Helmann, 2006). YknWXYZ shows homology to other ABC-type transporters such as MacAB-TolC of *E. coli*, and in addition to the MFP YknX, contains the components YknYZ and YknW (Figure 6). YknY and YknZ are an ATPase and a permease components of the transporter. YknW contains four transmembrane domains, and shares homology with the Yip1 family of proteins which are involved with vesicular transport in yeast. YknW was shown to affect the oligomeric state of YknX, or possibly YknX and YknYZ interactions (Yamada et al., 2012). This furthers the idea that the MFP-transporter interface is largely dependent on conformational changes within the MFP, and that stimulation of these changes can be conferred by proteins other than the transporter and OMF.

The functions of Gram-positive MFPs in transporter cycles await further studies. However, activities of Gram-negative MFPs are not limited to that of engaging an outer membrane channel and some of such activities could be also characteristic of Gram-positive MFPs. First, similar to Gram-negative MFPs, YknX and other Gram-positive MFPs are expected to be conformationally flexible. A wide range of MFPs movement (Mikolosko et al., 2006; Vaccaro et al., 2006; Wang et al., 2012) has been proposed to be important for stimulation of transporter activities, assembly of functional complexes and substrate extrusion. Second, MFPs of metal-exporting transporters are known to bind their substrates. The MFP ZneB from the *Cupriavidus metallidurans* ZneCAB export complex is known to exhibit structural changes upon binding its substrate zinc (De Angelis et al., 2010), the MFP CusB of the efflux complex CusBAC has been shown to bind its substrate, copper (Su et al., 2009; Delmar et al., 2013), and the MFP MacA binds a core LPS molecule, a putative substrate (Lu and Zgurskaya, 2013). It is possible that Gram-positive MFPs also receive substrates from their cognate transporters and then release them into the external medium. Finally, MFPs have also been proposed to stabilize or destabilize certain conformations of the transporter, moving the complex through the transport process. Hence, MFPs in Gram-positive cell envelopes, as in the context of Gram-negative cell envelopes, are likely to play an active role in the transport process. Interestingly, the predicted MFP of an ABC-type transporter DSY0927 from *Desulfitobacterium hafniense* is a fusion of two different MFPs. The N-terminal half of the protein shows homology to MF-type MFPs and does not have a coiled-coil domain, whereas the C-terminal half of the protein shares more characteristics of an RND-type MFP (Zgurskaya et al., 2009). This finding further suggests that similar to Gram-negatives, Gram-positive MFPs likely function as dimers, with each MFP molecule attaining a specific role in the transport cycle.

## Conclusions

The proposed model together with recent structural and functional advances provides a fairly detailed understanding of the mechanism of drug efflux across the two membrane envelopes of Gram-negative bacteria. Allosteric inhibitors that target specific conformations of MFPs and prevent these proteins from binding to OMF and activation of transporters could be effective in potentiation of activities of antibiotics. Further studies are needed: (i) to identify MFP-transporter and MFP-OMF molecular interfaces that are critical for activation of efflux and (ii) to characterize conformational transitions in all components of the complexes leading to efflux across the outer membrane. Non-typical transporters could be powerful tools in understanding how the trans-envelope efflux is achieved. There is a significant gap in knowledge about functions of MFPs in the context of Gram-positive cell envelopes.

### Conflict of interest statement

The authors declare that the research was conducted in the absence of any commercial or financial relationships that could be construed as a potential conflict of interest.
